# End Stage Kidney Disease Outcomes in Children and Young Adults with Sickle Cell Disease in the United States Renal Data System

**DOI:** 10.21203/rs.3.rs-2977181/v1

**Published:** 2023-05-26

**Authors:** Rima S. Zahr, Kenneth I. Ataga, Jeffrey D. Lebensburger, Jeffrey C. Winer

**Affiliations:** University of Tennessee Health Science Center College of Medicine Memphis; The University of Tennessee Health Science Center; University of Alabama at Birmingham; The University of Tennessee Health Science Center College of Medicine

**Keywords:** Transplant, Dialysis, Sickle Cell Disease, Outcomes

## Abstract

**Background:**

Children and young adults with sickle cell disease (SCD) develop kidney disease early in childhood with some patients progressing to require dialysis and kidney transplantation. The prevalence and outcomes of children with end stage kidney disease (ESKD) due to SCD is not well described. This study aimed to assess the burden and outcomes of ESKD in children and young adults with SCD in a large national database.

**Methods:**

Utilizing the United States Renal Data System (USRDS) we retrospectively examined ESKD outcomes in children and young adults with SCD from 1998 – 2019.

**Results:**

We identified 97 patients with SCD that developed ESKD and identified 96 matched controls with median age of 19 years (IQR 17, 21) at time of ESKD diagnosis. SCD patients had significantly shorter survival (7.0 years vs. 12.4 years, p < 0.001) and had a longer waiting time to their first transplant when compared to matched non-SCD-ESKD patients (10.3 years vs. 5.6 years, p < 0.001).

**Conclusions:**

Children and young adults with SCD-ESKD have a significantly higher mortality when matched to non-SCD-ESKD children and experience a longer mean time to kidney transplant.

## Introduction

Sickle cell disease (SCD) is an inherited hematologic disorder that causes progressive damage in many organs, including the kidneys. The prevalence of albuminuria, an early manifestation of chronic kidney disease, in children is high with around 10–30% of pediatric patients with sickle cell anemia (usually refers to homozygous SCD [HbSS] and sickle β° thalassemia [HbSβ°]) developing albuminuria and many patients transition from hyperfiltration to a normal filtration rate in kidney function during adolescence[1]. The literature on progression to ESKD in children is limited. A 1995 study from the National American Pediatric Renal Trials and Collaborative Studies (NAPRTCS) identified that only 0.5% and 0.2% of pediatric patients that progressed to require either dialysis or kidney transplantation, respectively, were in patients with SCD[2]. However, this data does not represent the true burden of disease as only US centers belonging to the collaborative entered their patient data and adoption. Importantly, since 1995, the acceptance of SCD modifying therapies has markedly improved and may impact the incidence of ESKD in pediatric SCD.

It is well-established that end-stage kidney disease (ESKD) contributes to the increased mortality in adults with SCD [3, 4]; however, the data on pediatric mortality from ESKD is limited. Childhood survival in the United States has improved for patients with SCD due to interventions that reduce acute mortality from infections; with this improved survival, mortality from chronic end-organ damage is increasing[4]. In adult individuals with SCD-ESKD who initiate dialysis, the one-year mortality after initiating dialysis is approximately three times higher in adult individuals without SCD but with ESKD [3]. Further, the risk of death in the first year of dialysis could be attenuated by more than 6 months with pre-dialysis nephrology care [3]. In pediatric individuals with SCD- EKSD, the survival outcomes and risk for premature death is not well established; this information is necessary to provide guidance for care teams. In order to evaluate the burden of SCD-ESKD, we performed a study of relative survival and time to transplant of children with ESKD using the United States Renal Data System (USRDS) registry. We hypothesized that survival of children with SCD-ESKD has improved over the course of the last two decades.

## Patients and Methods/Materials and Methods

We conducted a retrospective cohort study of children and young adults with a diagnosis of SCD-ESKD who had their first ESKD service aged 21 years and younger, between the years of 1998–2017 using data from the US Renal Data System (USRDS)[5]. The USRDS is a national data system that collects information on individuals with ESKD in the United States. We performed propensity matching for year of initial ESKD service, age, sex, race, and ethnicity between patients with SCD-ESKD and non-SCD-ESKD. Unmatched characteristics included first ESKD event modality type, pre-ESKD nephrology care duration, and comorbidities (congestive heart failure, cerebrovascular disease, and hypertension).

Demographics and initial therapy were compared between unmatched and matched groups using Chi-square or Fisher exact testing, as appropriate. Kaplan-Meier survival curves were created for the matched groups comparing survival and time to transplant between SCD-ESKD and non-SCD-ESKD. All statistical analysis was performed in SPSS (Version 27.0; IBM Corp, Armonk, NY). A p value of < 0.05 was considered significant. This study was approved by the institutional review board at the University of Tennessee Health Science Center at Memphis and USRDS. Per USRDS requirements all categories with fewer than 11 patients were censored.

## Results

We identified 97 (0.03%) children and young adults with SCD diagnosed with ESKD and 31,536 children and young adults with non-SCD-ESKD from 1998–2017 (Supplementary Table 1). Patients with SCD-ESKD were primarily young adults, with a median age of 19 years [IQR 17, 21]. Male patients comprised 67% of patients with SCD-ESKD.

We were able to identify 96 pairs with exact matches for initial ESKD year of service, age, sex, race, and ethnicity. We identified statistically significant differences between SCD-ESKD patients and unmatched non-SCD-ESKD patients for age, Black race, Hispanic ethnicity, first ESKD event modality type, pre-ESKD nephrology care, congestive heart failure, hypertension, and cerebrovascular disease. After matching, due to censoring patient numbers that were less than 11 per category no further associations were assessed.

**Figure 1:**
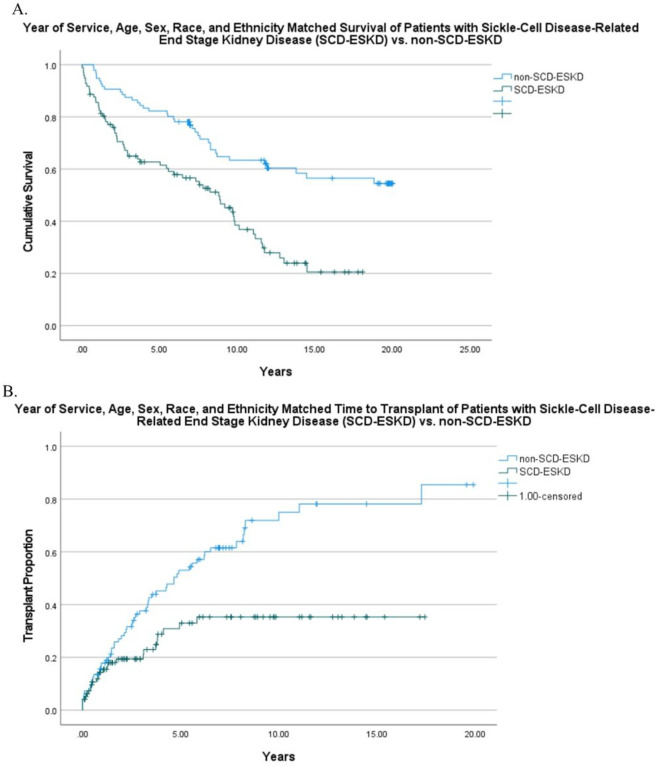
Comparison between year of service, age, sex, race, and ethnicity-matched patients with sickle-cell disease-end-stage kidney disease (SCD-ESKD) and non-SCD-ESKD. A) Kaplan-Meier mean survival time after ESKD diagnosis, 7.0 years vs. 12.4 years, p <0.001 and B) Kaplan-Meier time to transplantation, 10.3 years vs. 5.6 years, p < 0.00.

The mean survival time after ESKD diagnosis for children with SCD-ESKD patients (Fig. 1a) was significantly shorter than matched non-SCD-ESKD patients (7.0 years vs. 12.4 years, p < 0.001). SCD-ESKD patients experienced a longer mean time before receiving transplant (Fig. 1b) than matched non-SCD-ESKD patients (10.3 years vs. 5.6 years, p< 0.001).

## Discussion

This study further highlights disparities in health outcomes and access to transplant for children and young adults with SCD- ESKD. First, children and young adults with SCD-ESKD have worse survival than matched individuals with non-SCD-ESKD. Second, SCD individuals experience longer wait times for kidney transplant than matched controls. Third, despite adjusting for pre-nephrology care, children, and young adults with SCD-ESKD had overall higher mortality. The recent American Society of Hematology guidelines for the management of SCD suggests early referral for renal transplant and highlights the need for studies to evaluate disparities in access to care for SCD patients with ESKD[6]. This study provides additional evidence to support the need to further understand and address the reasons for this marked healthcare disparity.

Kidney transplantation in children is well-recognized to provide a long-term survival advantage in all patients with ESKD, however racial disparities in transplantation can lead to unequitable transplantation rates [7]. Two adult SCD studies suggest that transplantation is associated with a decrease in mortality [8, 9]. However, in this study, the time from the development of ESKD to kidney transplant remained longer for patients with SCD. This disparity is further highlighted by findings that these individuals have a higher mortality within the first and subsequent years after developing ESKD. Therefore, children, and young adults with SCD should not be precluded from early referral to access to kidney transplantation. A study published in 2021, examined the United Network for Organ Sharing (UNOS)/Organ Procurement and Transplant Network (OPTN) database, found that patient and allograft survival in SCD kidney recipients did not improve between recent era (2010–2019) and early era (2000–2009). However, the authors note that these findings should not discourage kidney transplantation due to the overall survival benefits that transplantation confers [10]. Several of these more recent studies point towards graft loss secondary to poorly controlled SCD-related disease. Strategies to reduce post-transplant complications due to SCD should therefore be evaluated. Disease modifying agents such as hydroxyurea, is both effective in reducing increased sickle hemoglobin concentration and decreasing albuminuria in children and adults with SCD [11] [12] and could be continued post-transplant. Initiating automated blood transfusion in adult recipients during and after kidney transplantation has also demonstrated the ability to improve allograft and patient outcomes [8]. The mammalian target of rapamycin (mTOR) pathway has been identified as a regulator of red blood cell growth and proliferation[13]; one case report found use of mTOR inhibitor, everolimus, post-transplant improved fetal hemoglobin and decreased SCD pain[14]. Lastly, allogenic hematopoietic stem cell transplantation remains the only curative therapy for SCD, but this can be limited by the cost, suitable matched donors as well as significant morbidity[15]. An approach of dual stem cell and organ transplantation may be an option for patients in the foreseeable future as advances in stem cell transplantation are made. Larger prospective studies are needed to confirm these medications and modalities on allograft survival.

Our study has several limitations inherent to a retrospective study of administrative data. We can only identify associations between SCD status and mortality/transplantation but cannot speak directly to causal relationships between delayed transplantation and mortality. We only identified SCD-ESKD individuals and cannot calculate the prevalence or incidence of ESKD within the SCD population at large. Additionally, due to the limited number of participants within the SCD-ESKD cohort and the small number of patients in many subgroups, we were unable to further stratify the data. We have limited information about care prior to developing ESKD, particularly regarding the use and duration of disease modifying therapies prior to development of ESKD and transfusions post-transplant. Future studies need to evaluate approved therapies for treatment of SCD to understand how these interventions influence the progression of kidney disease.

It is vital that the transplant community identify and reduce the barriers to kidney transplantation in children and young adults with SCD-ESKD. Future research should consider qualitative assessment of the barriers to transplantation among kidney transplant centers. Equitable access to transplantation may mitigate the decreased survival seen in this study. The risk of SCD-related kidney disease must be balanced with improvement in the quality of life and possible improved survival when compared to those who remain on dialysis. Use of hydroxyurea, mTOR inhibitors and exchange transfusions need to be further studied in the post-transplant period. However, in the immediate future, nephrology centers can start to work towards improving access to transplantation and establishing sickle cell specific transplant protocols to improve post-transplant allograft survival by working with their hematology colleagues and sharing protocols.

## Abbreviations

ESKD: End Stage Kidney Disease
SCD: Sickle Cell Disease
USRDS: United States Renal Data System
APOL1: Apolipoprotein L1
